# Towards Sustainable Temperature Sensor Production through CO_2_-Derived Polycarbonate-Based Composites

**DOI:** 10.3390/polym16131948

**Published:** 2024-07-08

**Authors:** Ane Martín-Ayerdi, Luis Rubio-Peña, Nikola Peřinka, Itziar Oyarzabal, José L. Vilas, Pedro Costa, Senentxu Lanceros-Méndez

**Affiliations:** 1 BCMaterials, Basque Center for Materials, Applications and Nanostructures, UPV/EHU Science Park, 48940 Leioa, Spain; ane.martin@bcmaterials.net (A.M.-A.); itziar.oyarzabal@bcmaterials.net (I.O.); joseluis.vilas@ehu.es (J.L.V.); 2 Engineering School, University of Cadiz, Avda. de la Universidad de Cádiz, 10, 11519 Puerto Real, Spain; luis.rubio@uca.es; 3 IKERBASQUE, Basque Foundation for Science, 48013 Bilbao, Spain; 4 Macromolecular Chemistry Group (LABQUIMAC), Department of Physical Chemistry, Faculty of Science and Technology, University of the Basque Country (UPV/EHU), Barrio Sarriena s/n, 48940 Leioa, Spain; 5 Physics Centre of Minho and Porto Universities (CF-UM-UP) and Laboratory of Physics for Materials and Emergent Technologies (LapMET), University of Minho, Campus of Gualtar, 4710-057 Braga, Portugal; pcosta@fisica.uminho.pt; 6 IB-S Institute of Science and Innovation for Sustainability, Universidade do Minho, 4710-057 Braga, Portugal

**Keywords:** polycarbonate, flexible electronics, carbon nanotubes, sensor, thermoresistive

## Abstract

The steep increase in carbon dioxide (CO_2_) emissions has created great concern due to its role in the greenhouse effect and global warming. One approach to mitigate CO_2_ levels involves its application in specific technologies. In this context, CO_2_ can be used for a more sustainable synthesis of polycarbonates (CO_2_-PCs). In this research, CO_2_-PC films and composites with multiwalled carbon nanotubes (MWCNTs, ranging from 0.2 to 7.0 wt.%) have been prepared to achieve more sustainable multifunctional sensing devices. The inclusion of the carbonaceous fillers allows for the electrical conductivity to be enhanced, reaching the percolation threshold (P_c_) at 0.1 wt.% MWCNTs and a maximum electrical conductivity of 0.107 S·m^−1^ for the composite containing 1.5 wt.% MWCNTs. The composite containing 3.0 wt.% MWCNTs was also studied, showing a stable and linear response under temperature variations from 40 to 100 °C and from 30 to 45 °C, with a sensitivity of 1.3 × 10^−4^ °C^−1^. Thus, this investigation demonstrates the possibility of employing CO_2_-derived PC/MWCNT composites as thermoresistive sensing materials, allowing for the transition towards sustainable polymer-based electronics.

## 1. Introduction

In recent years, there has been increasing concern about the steep increment in carbon dioxide (CO_2_) levels, which is associated with global warming and the greenhouse effect [1]. According to EDGAR, the Emissions Database for Global Atmospheric Research, CO_2_ emissions increased from 1990 to 2021 in the following manner: 87% due to the power industry, 65% due to other industrial combustion, 66% due to transport, 2% due to building-related emissions, and 101% due to emissions from other sectors. It is worth highlighting that in 2020, a reduction in CO_2_ emissions was recorded due to the COVID-19 pandemic. Nevertheless, in 2021, global emissions rebounded to reach 37.9 Gt, comparable to the levels observed in 2019 before the pandemic [2].

CO_2_ represents an abundant feedstock; therefore, several efforts have been made to harness the potential of this gas through recycling and reutilization, with the aim of bolstering the circular economy while concurrently mitigating its environmental footprint [3]. Some examples to accomplish the intended goal involve the use of CO_2_ in chemical production, including formic acid [4], urea [5], or in the synthesis of CO_2_-based polycarbonates (CO_2_-PCs) [6].

**Scheme 1:**
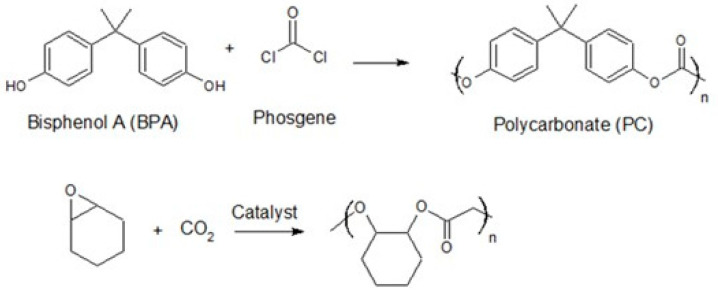
Polycarbonate synthesis from petroleum (above) and CO_2_ (below).

PCs are polymers composed of carbonate (-O-(C=O)-O-) monomeric units which are repeated along the polymer chain. The physical–chemical properties of this polymer—optical transparency, high thermal stability, and high tensile strength [7,8]—allow its application in several fields, including biomedicine [9], automotive [10], and electronics [11]. Petroleum-derived PCs are generally synthesized from bisphenol A (BPA), which usually improves PC resistance and durability [12], and phosgene. It has to be highlighted that these reagents are quite toxic. Phosgene is a poisonous gas, whereas BPA is a hormone and endocrine-disrupting chemical, as well as a neurotoxic and carcinogenic agent [13,14]. Consequently, green chemistry and environmentally friendly approaches to obtain PC monomeric units have been explored. An alternative relies on the copolymerization of CO_2_ and epoxide, which eliminates the need for toxic and environmentally harmful reagents such as phosgene and bisphenol A, as illustrated in Scheme 1 [15].

PCs have found extensive use in the electronics sector, primarily as an insulating layer. For instance, thermoplastic polycarbonate/acrylonitrile–butadiene–styrene blends (PC/ABS) have been incorporated into electronic devices, which impart not only insulating properties but also flame-retardant characteristics to the devices [16]. Additionally, PCs have been used as matrices to develop conductive composites. Even though they are dielectric polymers, electrical conductivity properties can be provided by adding conductive carbonaceous nanofillers to the matrix, including carbon nanotubes (CNTs) [17], graphene (G) [18], and carbon black (CB) [19]. In fact, one of the main approaches to obtain conductive PC composites involves the incorporation of CNTs, as they exhibit a high aspect ratio, large electrical conductivity values of 10^5^ S·cm^−1^, an elastic modulus of 1 TPa, and thermal conductivity values ranging from 3000 to 6000 W·mK [20,21]. Furthermore, the high aspect ratio of CNTs enables polymer/CNT hybrid materials to exhibit electrical conductivity above the percolation threshold (P_c_) at low CNT concentrations. The concentration depends on factors such as the nanotube morphology (single or multiwalled), processing method (extrusion or solvent casting), and dispersion quality [22]. Therefore, the dielectric polymeric matrix can be transformed into a conductive composite [23,24].

There is a wide option of polymers for composite processing. Nevertheless, a green transition to environmentally friendly electrically conductive composites is highly necessary. Interestingly, the field of biopolymer/nanofiller composites has led to numerous breakthroughs in recent years. Biopolymers are selected for their natural origin, abundance, renewability, and, in some cases, their ability to dissolve in water, which avoids the use of organic solvents [25]. All in all, these efforts support the required reduction in the environmental impact of the materials used in sensor and actuator applications [26,27].

For example, its natural origin and physical–chemical properties make silk fibroin a biopolymer to study. In this case, it has been reinforced by solvent casting with CNTs for piezoresistive sensor fabrication [28]. Other example includes graphene fillers dispersed into sodium carboxymethyl cellulose matrix by solvent casting for deformation sensing. The sensor has been processed following water-based formulation. This makes it attractive as the use of organic solvents is avoided. Even more, it shows thermoresistive sensitivity of S = −0.27 and piezoresistive Gauge Factors (GF) of 1 < GF < 5 [27]. Nonetheless, bio-based polymer composites still raise concerns with respect to sensor response stability over time.

Nevertheless, biopolymers are not the only way to achieve sensors following green chemistry. The upcycling of materials, the reuse of waste in other applications, is a suitable alternative to obtain high-performance materials while supporting sustainable approaches. In this scope, we suggest the development of PC-based sensing composites. This material has gained popularity due to the increasing demand for sensor materials driven by the digitalization of society and the Internet of Things concept. Consequently, numerous studies have explored the synthesis of PC/MWCNT composites as piezoelectric sensors. For instance, P. Costa et al. achieved composites with a P_c_ value of 0.3 wt.% and Gauge Factors between 1.1 < GF < 1.75 and 0.1 < GF < 0.4, respectively, for uniaxial strain and four-point-bending experiments, which could be applied in aeronautics [29]. Additionally, there have been significant advancements made in processing PC blends to enhance matrix characteristics and make them suitable for the aforementioned applications. For instance, P(VDF-HFP)/PC/MWCNT composites with piezoresistive sensing capabilities were prepared by melt spinning [30]. In the case of PC-CO_2_, there are different reports concerning their synthesis [31,32], as well as their physical–chemical characterization depending on chain length [33,34,35]. Nevertheless, there is a scarcity of literature concerning CO_2_-PCs combined with CNTs for sensing purposes. Consequently, the present study proposes CO_2_-derived PC/MWCNT composites for temperature sensor applications. Thermoresistive sensors rely on the variation in electrical resistance of the material with temperature, and the precise thermoresistive characteristics depend on factors such as the type and dispersion of the filler, filler dimensions, and specific chemical characteristics of both the polymer and the filler [36].

In summary, this investigation proposes an environmentally friendly approach for the fabrication of electronic materials and devices, as exemplified by the development of a thermoresistive sensing material. The approach involves the use of a poly (cyclohexene carbonate) (PCC) matrix synthesized from CO_2_ to develop composites with MWCNTs through a solvent casting process. The investigation includes the evaluation of the morphological, thermal, and electrical properties of the films, as well as their functional thermosensitive capabilities.

## 2. Materials and Methods

### 2.1. Reagents

Commercial QPAC^®^ 130 poly (cyclohexene carbonate) PCC was purchased as pellets from Empower Materials Inc., New Castle, DE, USA. The material shows a density of 1.10 g·cm^−3^, onset estimate decomposition temperature of 250 °C, and a glass transition temperature (T_g_) of 120–130 °C. As conductive fillers, multiwalled carbon nanotubes (MWCNTs) with reference NC7000™ were provided by Nanocyl, S.A, Sambreville, Belgium. MWCNTs were manufactured by chemical vapor deposition, showing a carbon purity of ≈90%, an average diameter of ≈9.5 nm, and an average length of ≈1.5 µm. Dichloromethane (DCM) was supplied from Sigma Aldrich (Burlington, MA, USA) and was used to dissolve the polymer and to disperse the MWCNTs.

### 2.2. Composite Films Processing

**Scheme 2:**
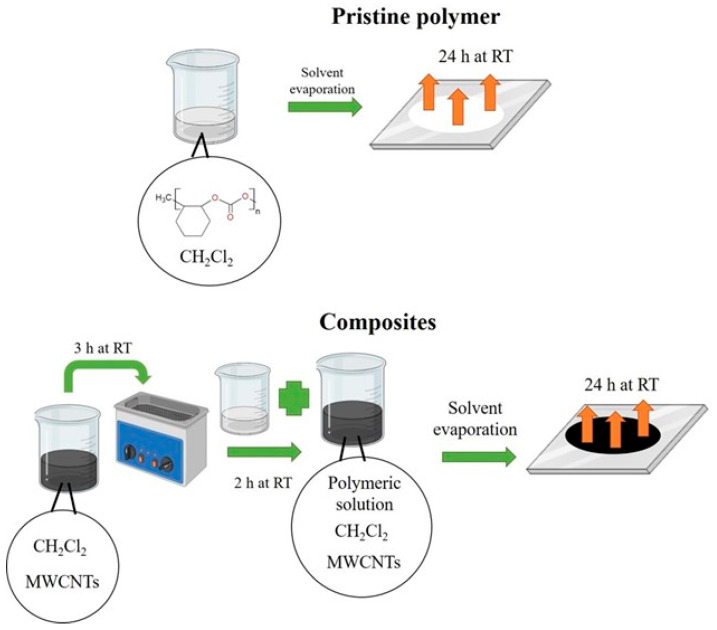
Sequential processing of pristine polymer and composites.

Pristine PCC films and PCC/MWCNT composites were prepared by the solvent casting method, as indicated in Scheme 2. Pristine PCC films were prepared by solving 2 g of polymer in 12 mL of DCM and stirring for 2 h at room temperature (RT). Composites were prepared in two steps. In the first step, 2 g of polymer was dissolved in 6 mL of DCM by stirring vigorously for 2 h at RT. Meanwhile, different amounts of MWCNTs (0.2, 0.5, 1.0, 1.5, 3.0, 5.0, and 7.0 wt.% with respect to the polymer) were weighted and 6 mL of solvent was added to them. The resulting suspensions were treated in an ultrasound bath for 3 h at 25 °C to improve deagglomeration and dispersion. In the second step, nanofillers were mixed with the polymer solution, and the resulting mixture was stirred for 2 h. In both cases, either with the pristine polymer or composite solutions, the solutions were poured onto a clean glass surface and films were subsequently produced using a Dr. Blade. Finally, the resulting films were dried for 24 h at RT to ensure solvent evaporation. After this process, films with an average thickness of 154 ± 27 µm were obtained.

### 2.3. Characterization

All samples were characterized in terms of morphology, thermal, mechanical, and electrical properties. The morphology of the samples was studied with a Hitachi S-4800 Scanning Electron Microscope (SEM) to evaluate the distribution of MWCNTs within the PCC matrix. Images were obtained at an accelerating voltage of 5.0 KV and magnifications of ×50.0K and ×100K.

Attenuated total reflectance–Fourier-transform infrared spectroscopy (ATR-FTIR) was used to analyze possible interactions between the polymer and the filler. Infrared spectra were collected with a Nicolet Nexus FTIR spectrophotometer (Thermo Electron Corporation, Waltham, MA, USA) in the range of 400 to 4000 cm^−1^ with a resolution of 4 cm^−1^ and averaging 64 scans per spectrum.

Thermal characterization was performed by Thermogravimetric Analysis (TGA) and Differential Scanning Calorimetry (DSC). TGA was measured in a Mettler Toledo TGA/SDTA851e thermobalance (Japan) to evaluate the maximum degradation temperature (T_max_). Samples (~10–15 mg) were weighted and heated from 25 to 800 °C at a heating rate of 10 °C·min^−1^ under a nitrogen atmosphere. For each sample, the maximum degradation temperature (T_dmax_) was calculated from the first derivative. Thermal transitions were measured with a Mettler Toledo model DSC 822e calorimeter (Greifensee, Switzerland). Samples (~10 mg) were sealed into aluminum pans and subjected to a temperature cycle ranging from −70 to 250 °C, followed by cooling from 250 to −70 °C. The thermal program consisted of successive heating, cooling, and heating scans, where the first scan was performed to remove the thermal history of the samples, while T_g_ was determined from the second heating scan.

The study of the mechanical properties allowed us to determine the initial modulus, maximum stress, and strain. All samples were measured in tensile mode using a Shimadzu AG-IS universal (Japan) testing machine equipped with a load cell of 500 N. Rectangular samples with approximate dimensions of 10 mm × 50 mm × 154 µm were tested at a constant velocity of 1 mm·min^−1^. Each sample was measured five times. The initial modulus was calculated up to 0.20% strain.

The electrical conductivity as a function of filler content and the corresponding percolation threshold (P_c_) were determined after measuring the samples with a Keithley 487 picoammeter/voltage source. Measurements were carried out by applying a voltage ranging from −10 V to +10 V with a step of 1 V in the direct current mode at RT and measuring the current. Prior to measurements, 5 mm diameter circular gold electrodes were deposited on both sides of the samples using a Quorum Q150T S sputter coater (Quorum Technologies, Kent, UK). The electrical conductivity of the samples was defined as the inverse of the resistivity (*ρ*), which was determined from the resistance (*R*) extracted from the I–V results following Equation (1), where *L* is the thickness of the sample and *A* is the area of the electrodes.

(1) $$\sigma =\frac{1}{\rho }=\frac{L}{RA}$$

Finally, thermoresistive tests were conducted to assess the suitability of the composites as temperature sensors. The thermoresistive performance of the PCC/MWCNT composites with 3.0 wt.% MWCNTs content was measured using an Agilent 34401A multimeter synchronized with a Linkam THMSE600 temperature oven. Silver ink (from Agar Scientific, Stansted Mountfitchet, UK, reference AGG3790) was used as a parallel conductive electrode, and electrical contact to the multimeter was performed with copper wires. The heating–cooling profile was divided into two different cycles, from 30 to 45 °C and from 40 to 100 °C.

(2) $$S=\frac{\Delta R/R_{0}}{\Delta T}$$

The thermoresistive sensitivity (*S*) was determined according to Equation (2), where Δ*R* and *R*_0_ are the electrical resistance variation and the initial resistance (in Ω), respectively, and Δ*T* is the temperature variation (in °C).

## 3. Results

### 3.1. Morphological and Chemical Characteristics

**Figure 1:**
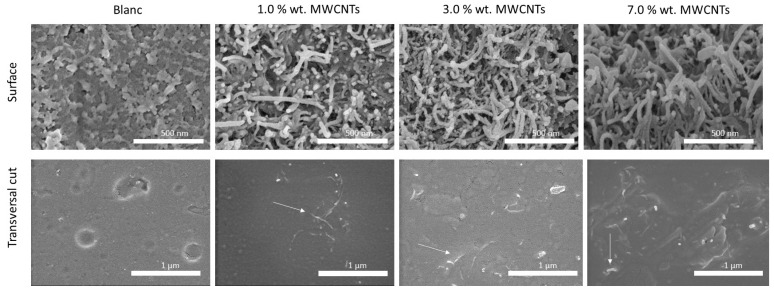
Surface (**above**) and cross-section (**below**) SEM images of PCC matrix and MWCNTs at 1.0 wt.% (**left**), 3.0 wt.% (**middle**), and 7.0 wt.% (**right**) filler content. White arrows point towards the MWCNTs, and clusters and agglomerates are dispersed within the matrix.

The morphology of the samples was evaluated by SEM images to analyze nanofiller dispersion within the matrix. Representative surface and cross-section images are shown in Figure 1. The pristine PCC film shows a flat surface and a compact morphology, though the presence of some voids is observed, which can be attributed to the solvent evaporation conditions. Regarding the films containing 1.0 wt.% MWCNTs, a good filler dispersion is observed together with a good compatibility (proper wetting with no voids around the fillers) between the polymeric matrix and the nanofiller (white arrows in Figure 1). Increasing the amount of MWCNTs from 1.0 to 3.0 wt.% leads to the presence of small clusters and agglomerates. Furthermore, the samples are less compact, showing a marked presence of voids, particularly in the cross-section images. Finally, the samples with 7.0 wt.% MWCNTs content show a large amount of well-distributed clusters along the samples (surface and cross-section images). The distribution of MWCNTs within the matrix affects both the electrical and mechanical properties, as clusters and agglomerates tend to bolster the electrical properties of the material more effectively than individual MWCNTs [37]. However, it is important to note that they often have a negative impact on the mechanical properties [38], as will be discussed later.

**Figure 2:**
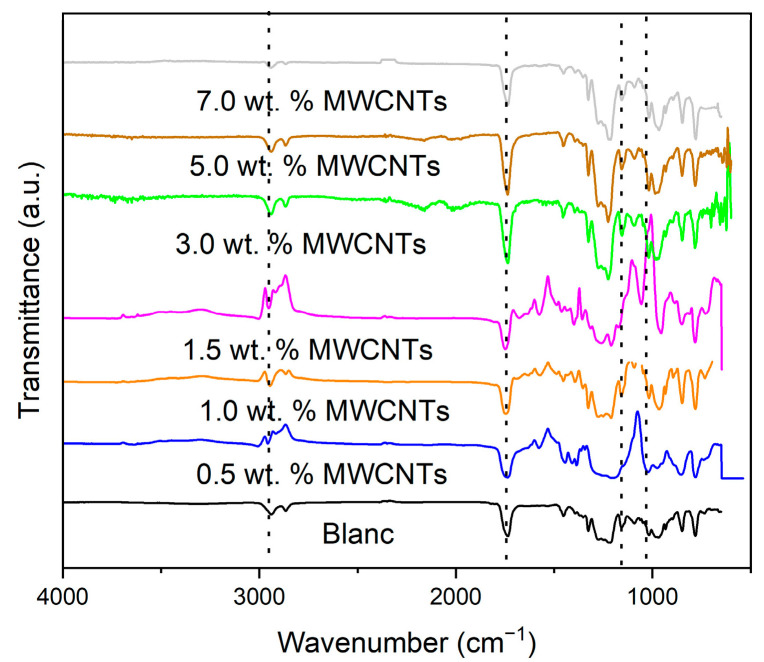
Infrared spectra of pristine PCC and PCC/MWCNT composites with different MWCNT contents.

The vibration spectra of the polymer and polymer composites and possible polymer–filler interactions were examined by analyzing the characteristic bands in the infrared spectra presented in Figure 2. In the case of pristine PCC, the main characteristic band is observed at 1735 cm^−1^, which corresponds to the carbonyl (C=O) stretching vibration. In addition, other bands related to the PC backbone are observed in the spectra: at 2945 cm^−1^, the C-H symmetric stretching vibration, at 1158 cm^−1^, the C-O-C asymmetric stretching vibration, and at 1015 cm^−1^, the symmetric O-C-O stretching [23]. Regarding the PCC/MWCNT composites, no significant band shifts are observed, indicating the absence of chemical interactions between the polymer and the filler [39,40].

### 3.2. Thermal Analysis

**Figure 3:**
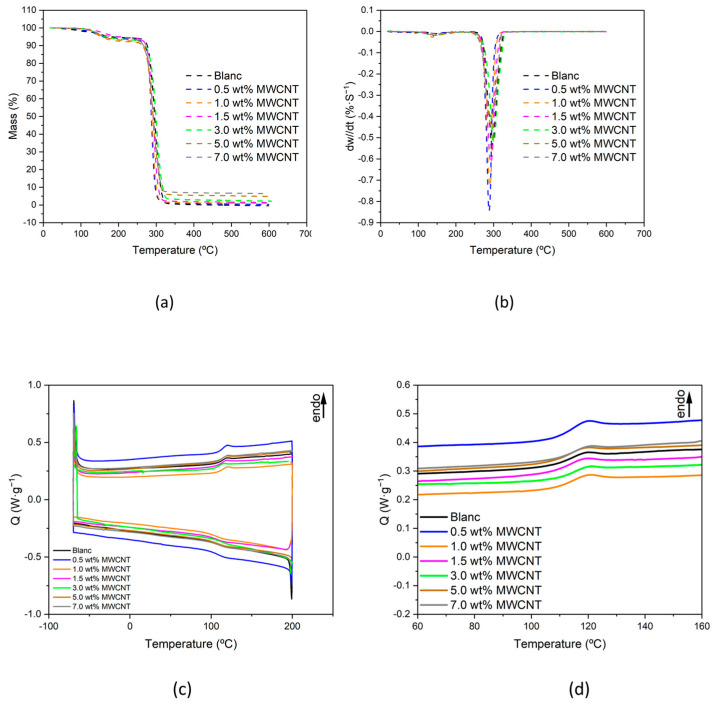
Thermal measurements of the blank sample and corresponding composites: (**a**) TGA curves, (**b**) the first derivatives, (**c**) DSC measurements, and (**d**) magnification between 70 and 150 °C temperature range to emphasize the T_g_.

The thermal behavior of PCC and composites is shown in Figure 3a up to 600 °C. The pristine polymer displays a single degradation step, associated with the pyrolysis of PCC carbonate groups [41]. Composites, alike the pristine sample, only depict one degradation step. After degradation, a small amount of residual weight is left, which increases linearly with the number of nanotubes added to the sample.

Thermal stability has been studied based on the first derivative of the TGA curves, as shown in Figure 3b. Maximum degradation temperature (T_dmax_) has been collected in Table 1. The polymeric matrix displays a thermal degradation temperature of 284.6 ± 9.3 °C, which does not suffer significant variations as a function of filler content, being the observed variations within experimental error.

The DSC results, depicted in Figure 3c,d, reveal a single thermal transition that is related to the glass transition temperature (T_g_) of the polymer. The glass transition temperatures at which the process starts (T_g-onset_) and finishes (T_g-offset_) are listed in Table 1. The T_g-onset_ values range from 110 to 112 °C, whereas the T_g-offset_ values are between 117 and 120 °C. All in all, these findings agree with previous thermal results, as they show that there is not a significant variation in the measured thermal characteristics of the samples due to the addition of the nanofillers [42]. The temperatures of the main thermal events are collected in Table 1.

### 3.3. Mechanical Properties

**Figure 4:**
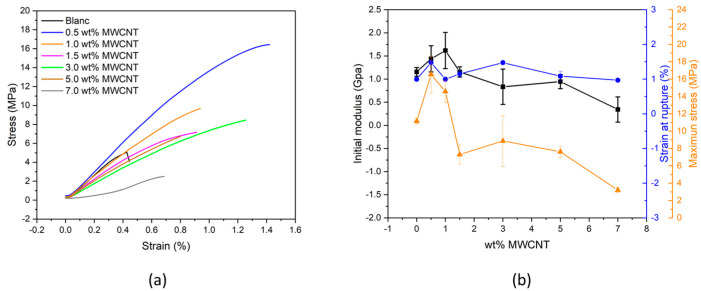
(**a**) Representative mechanical stress–strain characteristic curves of the different samples. (**b**) Calculated initial modulus, maximum strain, and stress parameters.

Mechanical properties were characterized by stress–strain experiments in the tensile mode (Figure 4a). These mechanical curves provide key parameters, including the initial modulus (E), as well as maximum strain and stress. Figure 4b shows the calculated initial modulus. The pristine PCC exhibits a value of EPCC = 1.16 ± 0.11 GPa. An increase in the amount of nanofiller leads to a higher initial modulus compared to the pristine PCC sample. This tendency is observed for nanofiller percentages up to 1.0 wt.% MWCNTs (E1.0% = 1.62 ± 0.37 GPa), but increasing the amount of filler from 1.5 wt.% to 7.0 wt.% decreases the initial modulus.

This behavior can be attributed to interactions between the MWCNTs, which lead to agglomerates and clusters, as well as to an increasing presence of voids in the films [37,43]. At low filler concentrations, the MWCNTs do not agglomerate, and the samples are more compact. However, as the amount of MWCNTs increases, agglomeration becomes more pronounced, reducing the interactions between the polymer and the filler. Furthermore, the structures are less compact and, therefore, more prone to deformation at lower stress. In addition to this, maximum stress and strain at break values (Figure 4b) were also determined. The maximum stress value decreases significantly with the inclusion of MWCNTs due to the mentioned agglomeration effect. Maximum strain values are ascribed to the brittleness of the composite.

### 3.4. Electrical Properties

From the relation between the electrical conductivity and filler percentage, the percolation threshold (P_c_) is calculated. This threshold provides the concentration at which a significant change in the electrical conductivity is observed, or in other words, the concentration at which the material changes its properties from dielectric to conductive. The P_c_ indeed depends on the intrinsic conductivity of the filler, its geometry, and its dimensional aspect ratio, among other parameters [44]. For example, PC/CNT composites prepared by the screw extruder method display P_c_ values of 0.125 wt.% [45]. Moreover, lower P_c_ values have been obtained for composites processed by the solvent casting method (0.06 wt.%) [42] and PC/PP blends prepared by the melt method (0.05 wt.%) [46].

**Figure 5:**
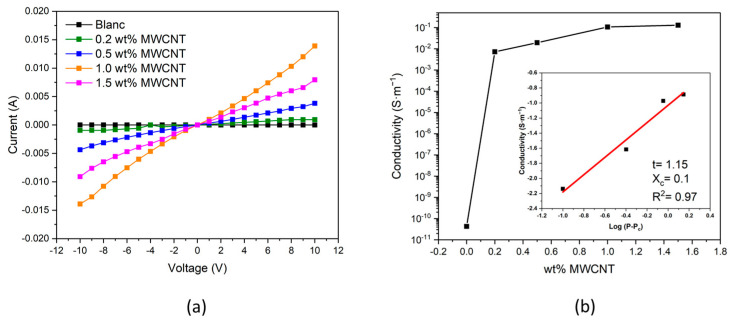
(**a**) Conductivity measurements based on current–voltage (I–V) curves; (**b**) electrical conductivity as a function of filler content. In black are the conductivity values obtained from the I-V curves and in red is the fitting based on the calculated P_c_ and *t* values.

Electrical properties were determined by current–voltage (I–V) measurement, as displayed in Figure 5a. Electrical conductivity was calculated using Equation (1). The obtained values as a function of the filler content are shown in Figure 5b. A maximum electrical conductivity of 0.107 S·m^−1^ was measured for the composite containing 1.5 wt.% MWCNTs. The percolation threshold of the samples was calculated following Equation (3), where σ is the composite’s electrical conductivity, $\sigma_{0}$ is the filler intrinsic conductivity, P is the filler concentration, P_c_ is the percolation threshold, and t is the dimensionality of the conductive network, being 1 < *t* < 1.3 for a 2D network and 1.5 < *t* < 2 for a 3D network [47,48].

(3) $$\sigma =\sigma_{0}{\left(P-P_{c}\right)}^{t}$$

The P_c_ and *t* parameters are fitted in Figure 5b. In the equation, P is the wt.% of MWCNTs. The best fit is obtained with P_c_ = 0.1 wt.% and *t* = 1.15 with R^2^ = 0.97. The exponential t parameter is compatible with the formation of a two-dimensional conductive network [42].

### 3.5. Thermoresistive Properties

The use of CO_2_-derived PC/MWCNT composites as sensors could contribute to reducing the CO_2_ footprint. While numerous publications have addressed PC/CNT composites as strain sensors, there is a scarcity of studies in the literature concerning their application as temperature sensors. Thus, this work studies the thermoresistive response (i.e., the change in electrical resistance (ΔR) as a function of temperature variation (ΔT)) [49] of CO_2_-PC/MWCNTs for temperature sensor applications.

**Figure 6:**
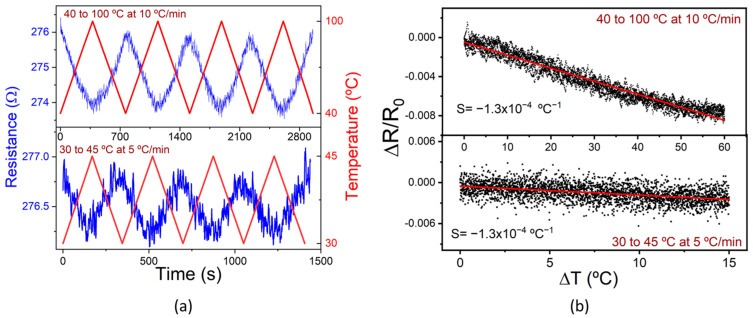
(**a**) Thermoresistance response for the composite containing 3.0% wt. MWCNTs and (**b**) the corresponding thermoresistive sensitivity.

In order to develop such a sensor, the composite requires a suitable electrical conductivity and, therefore, concentrations of nanofillers that exceed the P_c_ were selected. Consequently, the thermoresistive measurements were carried out using a conductive sample containing 3.0 wt.% MWCNTs. The thermoresistive properties were evaluated in two temperature regimes: from 30 to 45 °C, suitable for wearables and human body variations, and from 40 to 100 °C, for electronic devices, respectively. Both temperature ranges are depicted in Figure 6a, displaying an excellent correlation between resistance and temperature variations, although some minor electronic noise is noticeable mainly at lower temperatures. Even though the resistance changes are modest (approximately 1 and 2.5 Ω for ΔT of 15 and 60 °C, respectively), they follow the temperature profiles in both cases and are suitable for electronic readout systems.

The thermoresistive sensitivity (S) was determined using Equation (2), and the average of all cycles for each temperature profile is presented in Figure 6b. As previously mentioned, the sensitivity is approximately 1.3 × 10^−4^ °C^−1^ for both temperature ranges, respectively. While this value is not particularly high, it is high enough for electronic amplifications and transmission. In fact, the literature reports higher sensitivity values for petroleum-based polymer composites reinforced with CNT or graphene materials. However, in the present case, CO_2_-based polymer composites were employed, making a significant step towards more sustainable polymer-based electronic systems with reduced environmental impact.

## 4. Conclusions

This investigation reports on a more sustainable approach to develop polymer-based temperature sensors by employing CO_2_-PCC/MWCNT composites. The results revealed that increasing the concentration of nanofillers has a notable impact on the morphological, mechanical, and electrical properties of the nanocomposites, but not on their thermal properties. Regarding the mechanical assays, increasing the nanofiller content up to 1.0 wt.% MWCNTs results in an increase in the initial modulus (E1.0 = 1.62 ± 0.37 GPa). Above this concentration, a decrease in the initial modulus is observed due to the formation of agglomerates, which act as defects. However, these agglomerates enhance the electrical properties, reaching the electrical percolation threshold at P_c_ of 0.1 wt.%. Moreover, composites with nanofiller contents of 1.5 wt.% MWCNTs exhibit a maximum electrical conductivity value of 0.107 S·m^−1^. Among all the samples, the composite containing 3.0 wt.% MWCNTs emerged as the most suitable candidate for temperature sensing applications after considering its conductivity and mechanical properties. The thermosensitive test revealed a sensitivity of 1.3 × 10^−4^ °C^−1^ within the 40 to 100 °C and 30 to 45 °C temperature ranges. Even though this sensor exhibits a lower sensitivity than other petroleum-based polymer composites, it is worth noting that the sensor response is suitable for readout electronic systems. Therefore, it represents an important step towards more sustainable polymer-based electronic components based on CO_2_-derived and functional composites.

## Figures and Tables

**Table 1:** Summary of the temperatures of the main thermal events. T_dmax_ are obtained from TGA experiments, whereas T_g_ values are obtained from DSC.

wt.% MWCNT Content	TGA	DSC	
	T_dmax_	T_g-onset_ (°C)	T_g-offset_ (°C)
0	284.6 ± 9.3	111.2	118.3
0.5	279.4 ± 8.8	111.9	119.0
1.0	287.3 ± 5.9	112.1	118.6
1.5	286.3 ± 5.9	110.2	118.1
3.0	291.1 ± 7.8	111.3	117.4
5.0	286.5 ± 4.9	112.3	117.5
7.0	286.9 ± 6.3	111.7	120.0

## Data Availability

The original contributions presented in the study are included in the article, further inquiries can be directed to the corresponding author/s.
